# Role of the epitranscriptome in viral infections: beneficial or detrimental?

**DOI:** 10.1590/0074-02760250055

**Published:** 2025-10-03

**Authors:** Muhammad Fawwaz Abdullah, Kok Sing Yee, Nurhafiza Zainal, Sazaly AbuBakar, Chin Kim Ling

**Affiliations:** 1 Universiti Malaya, Faculty of Medicine, Department of Medical Microbiology, Kuala Lumpur, Malaysia; 2 Universiti Malaya, Tropical Infectious Diseases Research and Education Centre, Kuala Lumpur, Malaysia; 3 Universiti Malaya, Institute for Advanced Studies, Kuala Lumpur, Malaysia

**Keywords:** epitranscriptome, epitranscriptomic modifications, viral RNAs, virus-host interaction

## Abstract

Epitranscriptomics, the study of post-transcriptional chemical base modifications of RNAs, has become a crucial area of research for understanding the complex interactions between viruses and their hosts. These RNA modifications significantly impact both viral and host RNA functions, influencing viral replication, transcription, translation, and immune evasion. The advancement of high-throughput technologies, such as mass spectrometry-based techniques and next-generation sequencing, has enabled researchers to investigate epitranscriptomic modifications and their roles in gene regulation in greater depth. Viral RNAs often carry various epitranscriptomic modifications that facilitate their stability and translation, enabling viruses to hijack the host environment, enhance replication, and evade immune defences. Conversely, host epitranscriptomic modifications can enhance antiviral responses by regulating gene expression and promoting the degradation of viral RNAs. This dual role underscores the complexity of virus-host dynamics, where epitranscriptomic modifications can be both beneficial and detrimental. This review aims to provide an overview of current knowledge on epitranscriptomic modifications in viral infections, focusing on their roles in viral replication and immune interactions, while considering their potential as targets for antiviral therapeutic intervention.

Epitranscriptomic modifications, or RNA epigenetics, is a rapidly emerging field that focuses on the study of chemical base modifications of RNA molecules at the post-transcriptional level. These modifications play a crucial role in regulating gene expression by modulating various aspects of RNA metabolism, impacting cellular physiology and disease states, including cancers and neurological disorders.^1^ For instance, RNA modifications such as methylation are crucial post-transcriptional modifications that regulate RNA metabolism,^2^ nuclear export,^3^ exon splicing^4^ and translation.^5^ To date, more than 150 types of chemical modifications in cellular RNA have been identified in all types of RNA molecules, such as coding RNA like messenger RNA (mRNA); and non-coding RNA like ribosomal RNA (rRNA), transfer RNA (tRNA), long non-coding RNAs (lncRNAs), microRNA (miRNA) and small nuclear RNA (snRNA).^6^ ^,^ ^7^

MODOMICS, the first comprehensive database of RNA modifications, has recorded 180 nucleotide modifications, 152 nucleoside modifications, and three base-modifications.^6^ In general, epitranscriptomic modifications are regulated by a complex network of specialised protein enzymes called “writer”, that adds the modification; “reader”, that detects the modification and performs its phenotypic effects; and “eraser”, that removes the modification.^8^ However, common RNA modifications comprise mostly writer and reader proteins, which play crucial roles in many RNA modifications influencing many human biological metabolism and disease progression.^9^ Several common RNA modification sites have been recognised, such as N^6^-methyladenosine (m^6^A), 5-methylcytosine (m^5^C), 7-methylguanosine (m^7^G), N^1^-methyladenosine (m^1^A), N^4^-acetylcytidine (ac^4^C), adenosine-to-inosine (A-to-I) editing, 2’-O-methylation (2’-O-Me/Nm), pseudouridine (Ψ), uridylation, and phosphorylation.^10^

Although epitranscriptomic modifications have been extensively studied in human diseases, the role of epitranscriptomics in viral infection is less well understood. A previous report highlighted the importance of RNA modifications, particularly m^6^A, which significantly regulate viral gene expression and propagation.^11^ These RNA modifications play a crucial role in viral infection by influencing both the viral life cycle and the host’s immune response. In response to viral invasion, the host’s immune system typically develops countermeasures to restrict viral replication, which may involve epitranscriptomic modifications of antiviral genes.^12^ Retinoic acid-inducible gene 1 (RIG-I)-like receptors (RLRs) and toll-like receptors (TLRs) are the main pattern recognition receptors (PRRs). Their activation triggers specific antiviral signalling cascades, ultimately leading to the production of interferons (IFNs), IFN-stimulated genes (ISGs), and inflammatory cytokines, thereby limiting viral spread and orchestrating immune defence. Despite these host defences, viruses have evolved various mechanisms to exploit the host’s epitranscriptomic machinery to enhance their replication and persistence.^13^ Numerous modifications of viral RNA (vRNA) have been reported.^9^ ^,^ ^14^ For instance, m^6^A has been shown to stabilise vRNA and promote efficient translation of viral proteins.^15^ ^,^ ^16^ Therefore, these findings underscore the critical role of epitranscriptomic modifications in virus-host interactions, driving research interest due to their profound effects on viral replication, immune evasion, and pathogenesis.

This review aims to provide a comprehensive overview of recent advancements in the field of epitranscriptomic modifications in virus-host interactions, emphasising their dual roles in promoting viral replication and modulating antiviral responses in humans. Additionally, this review discusses potential antiviral therapeutic strategies that target RNA modifications, harnessing the therapeutic potential of the epitranscriptome in combating viral infections.

Epitranscriptomic modifications

mRNA transcripts are characterised by a 5’ cap modification, which affects transcript stability, pre-mRNA splicing, polyadenylation, mRNA export, and translation. Meanwhile, the poly(A) tail at the 3’ end facilitates nuclear export, translation initiation, recycling, and promotes mRNA stability through its association with the poly(A) binding protein family. The identification of cap and tail modifications has paved the way for the discovery of internal RNA modifications.

The mechanisms by which epitranscriptomic modifications regulate RNA-protein interactions can be summarised as follows: epitranscriptomic modifications can alter local RNA structures, which in turn affect the binding of RNA-binding proteins (RBPs).^17^ Alterations in RBP functions can disrupt multiple layers of gene regulation and cellular processes, potentially leading to significant biological consequences and disease states. For example, m^6^A modifications have been shown to influence IFN responses. The m^6^A writer, methyltransferase-like 3 (METTL3), adds m^6^A modifications to IFN mRNAs, enhancing the binding of YT521-B homology (YTH) domain-containing family protein 2 (YTHDF2), an RBP that promotes mRNA decay.^18^ This interaction plays a critical role in regulating IFN expression levels, which are crucial for antiviral responses. Loss of METTL3 or YTHDF2 leads to increased IFN expression and enhanced innate immune signalling, indicating that m^6^A modification can fine-tune immune responses against pathogens.

Various epitranscriptomic modifications serve as binding platforms for reader proteins that recognise the modified RNA and mediate downstream effects on gene expression. Reader proteins contain specialised domains that specifically bind to modified RNA, such as the YTH domain for m^6^A recognition.^19^ Epitranscriptomic modifications can also prevent the binding of anti-reader proteins that prefer unmodified RNA sequences. By blocking these anti-reader proteins, the modifications can indirectly regulate RNA-protein interactions and gene expression.^20^ Some modifications, such as inosine (I), can efficiently recode translation by preferentially base pairing with cytosine (C) instead of thymine (T).^21^ This property has been well established for inosine and enables the incorporation of non-cognate amino acids opposite inosine-containing codons.

Overall, epitranscriptomic modifications regulate gene expression by altering RNA structures, recruiting modification-specific reader proteins, preventing anti-reader protein binding, and modulating codon-anticodon pairing during translation. The dynamic interplay between writers, erasers, and readers allows for the fine-tuning of transcriptome plasticity in response to cellular and environmental cues. The most common RNA modifications are m^6^A, m^5^C, 2’-O-Me/Nm, m^1^A, m^7^G, Ψ, A-to-I editing, and uridylation. These modifications can be categorised into two main groups: RNA editing (Ψ and A-to-I editing) and methylation modifications (m^6^A, m^1^A, m^5^C, 2’-O-Me/Nm, m^7^G, and uridylation). Methylation modifications can further be classified based on the nucleotide involved: adenosine, cytosine, or guanosine.

Role of epitranscriptomic modifications in virus-host interactions

Epitranscriptomic modifications significantly influence how viruses interact with host cells. The study of these modifications in virus-host interactions reveals a complex interplay between epitranscriptomic changes and various biological processes, particularly in the context of viral infections and immune responses. These modifications play critical roles in how viruses invade the host and how host cells detect and respond to viral infections, shaping both innate and adaptive immune responses. In other words, epitranscriptomic modifications can serve both as a strategy employed by viruses to enhance their survival and replication and as a defence mechanism used by hosts. Several epitranscriptomic modifications have demonstrated either proviral effects (Table I) or antiviral effects (Table II), acting through distinct pathways that regulate various aspects of viral infection (Fig. 1) and modulate the host immune response (Fig. 2).

**TABLE I:** Proviral effects of RNA modifications

RNA modification	Proviral effects		References
	Viral replication	Innate immune responses	
m^6^A	Promote viral reproduction Enhance METTL3 protein (EV71, RSV) Enhance METTL14 (RSV) Enhance YTHDF2 protein (HIV-1) Enhance YTHDC1 protein (IAV) Enhance RIOK3 (DENV, ZIKV, HCV)	Inhibit host immune responses Evade RIG-1 recognition and inhibit IFN expressions (MPV, VSV, SeV, HBV, HCV) Enhance G mRNA (RSV) Inhibit IFN, TBK1, IRF3 (DENV, ZIKV, HCV)	^18^ ^,^ ^24^ ^,^ ^25^ ^,^ ^26^ ^,^ ^27^ ^,^ ^28^ ^,^ ^29^ ^,^ ^30^ ^,^ ^34^ ^,^ ^91^
m^5^C	Promote viral reproduction Enhance YBX1 protein (HCV) Enhance NSUN2 and DNMT2 (HIV-1) Enhance ALYREF (MLV, HBV)	Inhibit host immune response Inhibit RIG-1 binding and suppress IFN-β production (HBV) Suppress the binding of EGR-1 (HBV) Inhibit type I IFN response (RSV, MPV, VSV, SeV, HSV) Degrade *IRF3* mRNA (SARS-Cov-2, ZIKV, SeV, HSV-1)	^36^ ^,^ ^37^ ^,^ ^38^ ^,^ ^39^ ^,^ ^40^ ^,^ ^41^ ^,^ ^42^
2’-O-Me/Nm	Promote viral reproduction Enhance the stability and translation efficacy of vRNA (WNV, HIV-1) Mimic 2’-O-Me/Nm modification of host (SARS-CoV-2, MERS-CoV) Enhance vRNA binding affinity (SARS-CoV-2)	Inhibit host immune response Invade IFN-mediated antiviral response (SARS-Cov-2, WNV, DENV) Avoid detection of RIG-I and MDA5 through TRBP-FTSJ3 interaction (HIV-1) Degrade ISG20 (HIV-1)	^45^ ^,^ ^46^ ^,^ ^47^ ^,^ ^48^ ^,^ ^49^ ^,^ ^51^ ^,^ ^53^ ^,^ ^54^ ^,^ ^55^ ^,^ ^56^
m^1^A	Promote viral reproduction Utilise tRNA by using TRMT6 enzyme (HIV-1) Utilise tRNA by using TRMT61A enzyme (HIV-1, IAV)	ND	^57^ ^,^ ^58^ ^,^ ^59^ ^,^ ^60^ ^,^ ^61^ ^,^ ^62^
m^7^G	Promote viral reproduction Cap-snatching mechanism (IAV) Hypermethylate of m^7^G-cap by TGS1 (HIV-1)	Inhibit host immune response Utilise m^7^G cap with 2’-O methylation (IAV) Mimicking mTOR inhibition (HIV-1)	^61^ ^,^ ^63^ ^,^ ^64^ ^,^ ^65^ ^,^ ^67^
Ψ	Promote viral reproduction Enhance ncRNA, EBER2 (EBV) Upregulate BUD23 protein (KSHV) Enhance PUS7 (SARS-CoV-2) Stabilise host cofactor (HIV-1)	Inhibit host immune response Abolish PAMPs motif of RIG-I (SeV, VSV)	^69^ ^,^ ^70^ ^,^ ^71^ ^,^ ^72^ ^,^ ^73^
A-to-I Editing	Promote viral reproduction Editing-dependent and editing independent mechanism of ADAR1 (HIV-1) Enhance ADAR1 (DENV, ZIKV, SARS-CoV-2) Utilise ADAR to suppress DICER (SINV)	Inhibit host immune response Manipulate ADAR1 to inactivate PKR (ZIKV) Manipulate ACE2 (SARS-CoV-2, MERS-CoV) Manipulate host’s mRNA (DENV) Manipulate APOBEC and ADAR deaminases (SARS-CoV-2) Manipulate ADAR1 to inhibit IFN (HBV) Evade RLR and suppress *IRF3* (HBV, HCV, HIV-1, EBOV, MeV)	^75^ ^,^ ^78^ ^,^ ^83^ ^,^ ^84^
Uridylation	Promote viral reproduction Enhance vRNA stability (HAV, CMV) Regulate miR-122 (HCV) Enhance viral replication by using uridylate-specific endoribonuclease Nsp15 (SARS-CoV-2)	ND	^86^ ^,^ ^87^ ^,^ ^88^

N^6^-methyladenosine (m^6^A), 5-methylcytosine (m^5^C), 2’-O-methylation (2’-O-Me/Nm), N^1^-methyladenosine (m^1^A), 7-methylguanylate (m^7^G), Pseudouridine (Ψ), enterovirus 71 (EV71), human respiratory syncytial virus (RSV), human immunodeficiency virus 1 (HIV-1), influenza A virus (IAV), dengue virus (DENV), Zika virus (ZIKV), hepatitis C virus (HCV), human metapneumovirus (MPV), vesicular stomatitis virus (VSV), Sendai virus (SeV), hepatitis B virus (HBV), murine leukaemia virus (MLV), herpes simplex virus (HSV), severe acute respiratory syndrome coronavirus 2 (SARS-CoV-2), West Nile virus (WNV), Middle East respiratory coronavirus (MERS-CoV), Epstein-Barr virus (EBV), Kaposi’s sarcoma-associated herpesvirus (KSHV), Sindbis virus (SINV), EBOV (Ebola virus), measles virus (MeV), hepatitis A virus (HAV), human cytomegalovirus (CMV), not determined (ND).

**TABLE II:** Antiviral effects of RNA modifications

RNA modification	Antiviral effects		References
	Viral replication	Innate immune responses	
m^6^A	Suppress viral reproduction Inhibit YTHDF protein (HCV) Enhance METTL3 and METTL14 (HCV) Downregulate FTO (HCV) Enhance METTL3, YTHDF1, YTHDF3 (SARS-CoV-2)	ND	^18^ ^,^ ^29^ ^,^ ^33^
m^5^C	Suppress viral reproduction Enhance NSUN2 (EBV)	ND	^43^ ^,^ ^44^
2’-O-Me/Nm	ND	ND	ND
m^1^A	Suppress viral reproduction Upregulate of host m^1^A (SARS-CoV-2)	ND	^62^
m^7^G	ND	ND	ND
Ψ	ND	ND	ND
A-to-I Editing	Suppress viral reproduction Activation of PKR (HCV) Edit non-encapsidated defective interfering RNA (MeV) Edit binding ability of ACE2 (SARS-CoV-2)	ND	^80^ ^,^ ^81^ ^,^ ^82^
Uridylation	Suppress viral reproduction Interfere with vRNA stability and transcription (MHV, IAV)	Increase host immune response Enhance host’s mRNA decay pathways (IAV)	^89^ ^,^ ^90^

N^6^-methyladenosine (m^6^A), 5-methylcytosine (m^5^C), 2’-O-methylation (2’-O-Me/Nm), N^1^-methyladenosine (m^1^A), 7-methylguanylate (m^7^G), Pseudouridine (Ψ), hepatitis C virus (HCV), severe acute respiratory syndrome coronavirus 2 (SARS-CoV-2), Epstein-Barr virus (EBV), measles virus (MeV), mouse hepatitis virus (MHV), influenza A virus (IAV), not determined (ND).

**Fig. 1:**
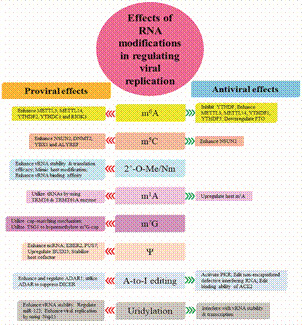
summary comparison of the effects (proviral and antiviral) of specific RNA modifications across different viruses on viral replication.

**Fig. 2:**
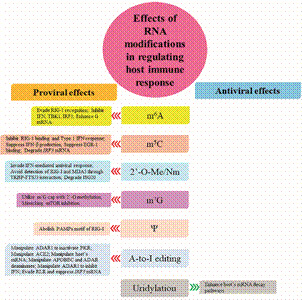
summary comparison of the effects (proviral and antiviral) of specific RNA modifications across different viruses on host immune response.

While the primary emphasis of this review is on mRNA modifications in viral infections, relevant findings on RNA modifications in non-coding RNA species, including miRNAs and lncRNAs, are also briefly considered, as these molecules are gaining increasing attention in the field.

*N* ^*6*^ *-Methyladenosine (m* ^*6*^ *A) modification* - m^6^A modification is the most abundant and prevalent internal RNA modification in many different eukaryotic species and viruses. The m^6^A modification process is dynamically orchestrated by methyltransferases/writers, such as METTL3 (a catalytic subunit) and METTL14 (an allosteric activator), which are responsible for adding the methyl group to adenosine residues in RNA;^22^ demethylases/erasers, such as AlkB homolog 5 (ALKBH5) and fat mass and obesity-associated protein (FTO), which reverse the modification;^23^ and proteins that recognise and bind to m^6^A-modified RNA (readers), such as the YTH domain family, which influence RNA stability and translation, including YTHDF1, YTHDF2, YTHDF3, YTHDC1, and YTHDC2.^11^

m^6^A modification is commonly identified in vRNAs and plays multiple virus-specific roles in replication processes. m^6^A promotes the replication of several viruses, such as enterovirus 71 (EV71),^24^ human respiratory syncytial virus (RSV),^25^ human metapneumovirus (MPV),^26^ influenza A virus (IAV),^27^ Sendai virus (SeV), vesicular stomatitis virus (VSV),^28^ dengue virus (DENV), Zika virus (ZIKV), hepatitis C virus (HCV),^29^ and human immunodeficiency virus 1 (HIV-1).^30^ For example, m^6^A in EV71 RNA can positively regulate METTL3 to enhance viral replication,^24^ while both m^6^A writer proteins, METTL3 and METTL14, facilitate RSV replication and gene expression.^25^ Moreover, m^6^A enhances HIV-1 replication by increasing mRNA stability and translation efficiency through the recruitment of the YTHDF2 protein,^30^ whereas YTHDC1 enhances IAV replication by inhibiting splicing of the nonstructural protein (NS) segment.^31^ Furthermore, m^6^A enhances the replication of DENV, ZIKV, and HCV by promoting RIO Kinase 3 (RIOK3), which facilitates vRNA translation and viral replication.^29^ Thus, these vRNAs can manipulate the m^6^A modification machinery to enhance their replication, creating a more favourable environment for virus propagation.^32^

On the other hand, m^6^A can also have antiviral effects, depending on the specific circumstances of the viral infection and the host’s response. For instance, m^6^A modification of vRNA acts as a suppressive regulator of viral replication in HCV by inhibiting YTHDF proteins involved in vRNA translation and stability, while the replication of HCV is negatively regulated by METTL3 and METTL14.^18^ The antiviral role of m^6^A is also demonstrated by the overexpression of the m^6^A demethylase FTO, which positively regulates HCV by promoting viral particle production.^18^ This indicates that demethylation of m^6^A enhances HCV replication, highlighting a complex interplay where increased levels of m^6^A suppress HCV replication, while its removal through demethylation facilitates viral particle production. Moreover, m^6^A modification has also been found in severe acute respiratory syndrome coronavirus 2 (SARS-CoV-2), where overexpression of METTL3, YTHDF1, and YTHDF3 suppresses viral replication.^33^

The impact of m^6^A on the immune response is profound, as viruses often exploit m^6^A modification to evade host immune detection. During viral infections, RLRs are crucial for recognising foreign RNA. However, several viruses use m^6^A-modified vRNAs to make them less recognisable by PRRs and avoid detection by innate immunity. The RNA genomes of MPV, VSV, SeV, hepatitis B virus (HBV), and HCV can evade detection by the RNA sensor RIG-I and suppress various IFN expressions, while HBV and HCV vRNA also exploit host m^6^A machinery to modulate METTL3 and METTL14, further inhibiting RIG-I recognition.^26^ ^,^ ^28^ ^,^ ^34^ Moreover, research has demonstrated that m^6^A modification in RSV enhances the stability and translation efficiency of G mRNA, thereby facilitating the virus’s escape from host innate immune surveillance.^25^ However, further clarification is needed to fully understand this mechanism of action. In addition, viruses can potentially dampen the host’s antiviral response through m^6^A modification of RIOK3, as it stimulates interactions between antiviral type 1 IFN, TANK-binding kinase 1 (TBK1), and IFN regulatory factor 3 (IRF3), which are critical components of the innate immune signalling pathway.^29^

In summary, m^6^A modification can exhibit both antiviral and proviral effects, contingent upon the specific virus and context. The m^6^A modification serves as a sophisticated regulatory mechanism in virus-host interactions, impacting both viral life cycles and host immune responses. Understanding these dynamics offers potential avenues for developing antiviral strategies targeting m^6^A pathways.

*5-Methylcytosine (m* ^*5*^ *C) modification* - 5-methylcytosine (m^5^C) methylation is an RNA modification occurring at the 5th carbon atom of cytosine (C), catalysed by methylases such as the NOP2/Sun RNA methyltransferase family (NSUN1-7) and DNA methyltransferase 2 (DNMT2). Conversely, m^5^C demethylases, including the ten-eleven translocation methylcytosine dioxygenases (TET1-3) and ALKBH1, can reverse this methylation. These methylated RNAs affect RNA export, transport, translation, and stabilisation through recognition proteins (readers) such as Aly/REF export factor (ALYREF) and Y-box binding proteins (YBX1-2).^35^

m^5^C methylation has been identified in several vRNAs, although its biological functions remain to be fully elucidated. Most viruses appear to require m^5^C modification mediated by host enzymes. This modification can exhibit both proviral and antiviral functions, depending on the specific viral context and host-virus interactions.

Many viruses harbour m^5^C-modified RNA, including Sindbis virus (SINV), canine adenovirus 2 (CAV-2), murine leukaemia virus (MLV), HIV-1, RSV, MPV, VSV, SeV, herpes simplex virus (HSV), HBV, HCV, and SARS-CoV-2.^36^ ^,^ ^37^ ^,^ ^38^ Viruses exhibiting higher levels of m^5^C modification in their RNAs may gain a replicative advantage compared to those with lower levels of this modification. For example, HCV infection induces the expression of the host m^5^C reader YBX1 through the transcription factor myc-associated factor X (MAX), which in turn facilitates viral replication by interacting with m^5^C-modified HCV RNA.^39^ Similarly, HIV-1 actively recruits NSUN2 and DNMT2 to modulate its replication,^40^ ^,^ ^41^ while MLV and HBV use the ALYREF reader protein to enhance viral replication efficiency.^36^ ^,^ ^42^ Hence, by modulating RNA stability and translation, m^5^C modifications contribute to the overall regulation of the viral life cycle, ensuring timely production of viral components necessary for successful replication and propagation. Conversely, Epstein-Barr virus (EBV) exhibits opposite effects, where downregulation of the host m^5^C methyltransferase NSUN2 leads to increased levels of vRNA in vivo, suggesting that m^5^C modification negatively impacts EBV vRNA replication.^43^

m^5^C RNA modifications also play a crucial role in controlling the innate immune response to viral infection. m^5^C modification at specific sites in HBV is essential not only for ALYREF recognition to promote viral mRNA export and hepatitis B viral protein (HBx) translation but also for inhibiting RIG-I binding to suppress IFN-β production.^36^ Moreover, NSUN2 catalyses the addition of m^5^C to HBV mRNA and is transcriptionally downregulated by HBx, which suppresses the binding of early growth response 1 (EGR-1)-a transcription factor involved in humoral immune response and regulation of IFN production-to the NSUN2 promoter.^36^ Furthermore, depletion of NSUN2 significantly enhances type I IFN responses against RSV, MPV, VSV, SeV, and HSV,^38^ while knockdown or knockout of NSUN2 enhances the expression of type I IFN and ISGs during viral infections with SARS-CoV, SeV, HSV-1, and ZIKV.^44^ Additionally, NSUN2 has been shown to be a negative regulator of type I IFN responses during viral infections by degrading *IRF3* mRNA; its knockout or knockdown enhances *IRF3* mRNA and protein levels, thereby amplifying IFN responses.^44^

In summary, m^5^C RNA modification is integral to the regulation of antiviral innate immunity, influencing both host and viral RNA dynamics. It affects RNA stability and immune evasion strategies employed by viruses, highlighting its role in facilitating viral replication and modulating innate immune responses. The dual role of m^5^C in supporting viral survival and regulating host defences underscores its complexity and significance. Ongoing research into m^5^C modifications is essential for understanding their multifaceted roles in immune responses and for developing potential antiviral therapies.

*2’-O-Methylation (2’-O-Me/Nm) modification* - 2’-O-methylation (2’-O-Me) or Nm (where N represents any nucleotide) is one of the most common RNA modifications in eukaryotes. Cellular mRNAs can be methylated at the 2’O sites of the 5’-guanosine cap by methyltransferases (MTases), acting as a molecular marker for the host immune system to differentiate self-RNA from non-self-pathogen RNA, which typically lacks 2’-O-Me/Nm.

The functions of 2’-O-Me/Nm in vRNA are multifaceted and essential for viral success. Several RNA viruses have been reported to incorporate this modification into their genomes, affecting the ribose component of RNA. West Nile virus (WNV) uses its own 2’-O-Me/Nm modification to enhance the stability and translational efficacy of vRNA by protecting it from degradation by host exonucleases.^45^ Moreover, the presence of the 2’-O-Me/Nm group at the 5’ cap structure makes the RNA of HIV-1 less susceptible to enzymatic cleavage, thus prolonging its half-life within the host cell.^46^ In some cases, the vRNA of SARS-CoV-2 and Middle East respiratory syndrome coronavirus (MERS-CoV) can mimic the 2’-O-Me/Nm modifications found on host mRNAs, enabling efficient recognition and translation by the host’s ribosomes.^47^ Furthermore, 2’-O-Me/Nm modification significantly improves the translation efficiency of SARS-CoV-2 viral proteins by enhancing vRNA binding affinity to 2’-O-methyltransferase (2’-O-MTase), improving its compatibility with the host’s RNA substrate.^48^ ^,^ ^49^ This increased stability is important for efficient viral replication, ensuring that the viral genome remains intact for translation and production of viral proteins necessary for the assembly of new virions within the host. Notably, SARS-CoV-2 can be reverse-transcribed and integrated into the genome of infected cells, resulting in persistent vRNA production even after patient recovery.^50^

2’-O-Me/Nm vRNA can modify host immune gene expression and downregulate the host’s translational machinery, potentially leading to altered immune responses.^51^ Viruses like SARS-CoV-2 and WNV utilise 2’-O-Me/Nm to evade the IFN-mediated antiviral response.^52^ Mutants lacking 2’-O-methyltransferase activity have been shown to exhibit increased sensitivity to IFN and enhanced immune response activation.^53^ Moreover, DENV lacking 2’-O-Me/Nm in its genome is attenuated and immunogenic during the early stages of infection, highlighting the essential role of this modification in immune evasion.^54^ The presence of 2’-O-Me/Nm allows vRNA to evade activation of antiviral pathways. This modification is crucial for HIV-1, as it enables the virus to evade detection by PRRs such as RIG-I and melanoma differentiation-associated protein 5 (MDA5). HIV-1 achieves this modification by recruiting the host’s FtsJ RNA 2’-O-methyltransferase 3 (FTSJ3) through the TAR-RNA-binding protein (TRBP) complex.^55^ Moreover, FTSJ3-knockdown cells have been shown to be more vulnerable to ISG20-mediated degradation, contributing to impaired reverse transcription and increased immune recognition.^56^

2’-O-Me/Nm plays a crucial role in virus-host interactions by enabling viruses to evade immune detection through modifications that mimic host RNA, thereby suppressing antiviral responses and facilitating replication. Additionally, 2’-O-Me/Nm modifications enhance the stability and expression of vRNA while modulating the host’s immune response, highlighting their significance in both viral pathogenesis and potential therapeutic strategies.

*N* ^*1*^ *-Methyladenosine (m* ^*1*^ *A) modification* - N^1^-methyladenosine (m^1^A) is a conserved modification involving the methylation of the N^1^ position of adenosine in eukaryotic non-coding RNAs. m^1^A shares several regulatory proteins with m^6^A, such as FTO, which acts as an eraser.^57^

m^1^A modification in vRNAs plays a significant role in viral replication, particularly in viruses like HIV-1 and IAV. The m^1^A modification in HIV-1 is primarily located in tRNA, which the virus utilises during reverse transcription.^58^ This modification is crucial for both the early and late stages of HIV-1 replication, as it enhances vRNA stability and replication efficiency. Enzymes such as the tRNA methyltransferases TRMT61A and TRMT6, key components of the m^1^A writer complex, add m^1^A to cytoplasmic tRNAs that serve as primers for synthesising viral DNA.^59^ The presence of m^1^A likely facilitates reverse transcription by providing a stable primer, thereby influencing the efficiency of HIV-1 replication.^60^ In addition, knocking down TRMT61A leads to reduced viral titres of IAV, suggesting that m^1^A modifications enhance the virus’s ability to replicate within host cells.^61^ Nonetheless, upregulation of m^1^A in host RNA can inhibit the viral replication complex of SARS-CoV-2,^62^ suggesting a dual role of m^1^A in viral and host contexts.

The m^1^A modification plays a crucial role in virus-host interactions by both inhibiting viral genome replication and stabilising vRNA. However, there is no evidence to show that m^1^A modification upregulates or helps evade host immune responses. Further studies are necessary to fully elucidate how m^1^A affects viral life cycles and its implications for treatment strategies against viral infections.

*7-Methylguanylate (m* ^*7*^ *G) modification* - 7-methylguanylate (m^7^G) is a crucial modification that occurs at the 5’ end of eukaryotic mRNAs. This cap structure is formed by the addition of a methyl group to the guanine nucleotide, creating a 7-methylguanylate cap. This modification is essential for various aspects of eukaryotic RNA metabolism, including stability, translation, and regulation of gene expression.

The m^7^G cap is essential for the stability and translation of IAV vRNA, facilitating efficient viral replication.^61^ ^,^ ^63^ This is accomplished through a cap-snatching mechanism, where a short, capped fragment of host RNA is utilised to initiate the synthesis of vRNA.^64^ Furthermore, HIV-1 can evade host restrictions through hypermethylation of the m7G cap using trimethylguanosine synthase 1 (TGS1), significantly promoting posttranscriptional expression of vRNAs.^65^ A previous study also found that one of the m^7^G writers, methyltransferase-like 1 (METTL1)/WD repeat domain 4 (WDR4), was downregulated after IAV infection. However, this downregulation did not affect viral growth after knockdown.^61^

Moreover, hypermethylation of HIV-1 m^7^G-cap mRNA, particularly those modified to trimethylguanosine (TMG), engages specific translation pathways that are less affected by mammalian target of rapamycin (mTOR) inhibitor signalling, which is often involved in the host’s immune response.^65^ Viruses such as IAV mask their RNA with the m^7^G cap and 2’-O-Me/Nm modifications, preventing recognition of vRNAs by RLRs, which are crucial for initiating the innate immune response.^63^ ^,^ ^66^ By masking the vRNA, the m^7^G cap can inhibit the activation of signalling pathways that would normally lead to the production of IFNs and other antiviral responses. Moreover, a recent report demonstrates that most m^7^G-related genes were upregulated in COVID-19 patients and closely associated with immune cell infiltration.^67^ However, further studies are needed to better understand the mechanisms and assess the impact of m^7^G on virus-host interactions.

*Pseudouridine* (Ψ) modification - The process of pseudouridylation involves the action of pseudouridine synthase (PUS) enzymes, which convert uridine to pseudouridine (Ψ).^68^ This modification can alter the base-pairing properties of RNA without changing its sequence, enabling the virus to evade detection during critical processes like reverse transcription.

Ψ residues can influence splicing and translation, potentially regulating gene expression to favour viral replication.^9^ A study has demonstrated that pseudouridylation of EBV noncoding RNA, EBV-Encoded RNA 2 (EBER2), is crucial for the accumulation of progeny viral genomes, underscoring its role in efficient viral replication.^69^ Similarly, Kaposi’s sarcoma-associated herpesvirus (KSHV) leverages pseudouridylation by regulating ribosomal subunit factor 23 (BUD23) to enhance replication and transcription complexes.^70^ In SARS-CoV-2, substantial Ψ modification mediated by PUS7 occurs during interactions with host cells, impacting the viral life cycle.^71^ Additionally, pseudouridylation stabilises a host cofactor required for HIV-1 transcription, thereby controlling both viral and host gene expression.^72^ Ψ modification has also been shown to regulate innate immunity. During SeV and VSV infection, Ψ modification abolishes filament formation of RIG-I and its binding to pathogen-associated molecular pattern (PAMP) motifs, leading to a reduced immune response.^73^

Unlike other modifications, Ψ is exclusively proviral. Its incorporation into vRNA stabilises RNA, enhances translation, and enables immune evasion, driving increased virulence and persistence. Understanding the mechanisms behind Ψ incorporation opens avenues for therapeutic interventions targeting PUS enzymes or disrupting Ψ processes, potentially leading to reduced viral loads and better outcomes for infected individuals.

*Adenosine-to-Inosine (A-to-I) editing modification* - A-to-I editing refers to the enzymatic conversion of adenosine (A) residues to inosine (I) in RNA molecules. This modification is catalysed by adenosine deaminases acting on RNA (ADARs), a family of enzymes that recognise double-stranded RNA (dsRNA) structures. Inosine is interpreted as guanosine (G) during translation, leading to potential changes in the coding sequence of the RNA.^74^

A-to-I editing can have proviral, antiviral, or neutral effects. A-to-I editing in HIV-1 vRNA can influence viral replication, with evidence showing that ADAR1 stimulates HIV-1 replication through both editing-dependent and editing-independent mechanisms.^75^ Similarly, ADAR1 can promote viral infectivity of DENV^76^ and SARS-CoV-2,^77^ and increase RNA synthesis, protein levels, and viral titres of ZIKV.^78^ In addition, SINV utilises ADAR to suppress endoribonuclease Dicer (DICER), a ribonuclease that cleaves dsRNA into small interfering RNAs (siRNAs), which are vital for gene regulation and antiviral defence.^79^ Conversely, A-to-I editing by ADAR1 reduces HCV replication by inhibiting vRNA translation through protein kinase RNA-activated (PKR);^80^ diminishes viral infectivity in measles virus (MeV) by editing non-encapsidated defective interfering RNA;^81^ and reduces SARS-CoV-2 transmissibility by altering angiotensin-converting enzyme 2 (ACE2) receptor binding ability.^82^

Furthermore, by modifying the coding sequence, A-to-I editing can regulate the expression levels of viral proteins, which is crucial for the virus’s ability to adapt to varying host environments and immune pressures. RNA A-to-I editing utilises the enzyme ADAR1 to modulate the activity of PKR in ZIKV,^78^ ACE2 in SARS-CoV-2 and MERS-CoV,^83^ and host mRNA in DENV,^76^ thereby regulating host innate immune responses. In addition, A-to-I editing mediates apolipoprotein B mRNA editing catalytic polypeptide-like (APOBEC) and ADAR deaminases in SARS-CoV-2 to evade immune responses.^83^ Furthermore, loss of ADAR1 or its deficient deaminase activity has been shown to increase IFN responses and inhibit HBV replication in hepatocytes.^84^ Additionally, A-to-I editing in HBV, HCV, HIV-1, Ebola virus (EBOV), and MeV can evade RLR detection and suppress IRF3, potentially influencing the virus’s ability to evade immune detection.^84^ ^,^ ^85^

In summary, A-to-I RNA editing significantly influences virus-host interactions by modulating viral replication and immune responses, with effects that can be either proviral or antiviral depending on the specific context. This editing mechanism alters the recognition of vRNA by host immune receptors, thereby impacting the activation of innate immune pathways and ultimately shaping the host’s antiviral response.

*Uridylation modification* - Uridylation, the addition of uridine residues to RNA molecules, plays a primary role in viral replication by stabilising vRNA. Many viruses have evolved specific strategies to exploit uridylation for their own benefit. Viruses such as hepatitis A virus (HAV) and human cytomegalovirus (CMV) utilise uridylation to enhance the stability of their RNA.^86^ Moreover, viral infection can regulate miRNA abundance by modulating the activity of cellular enzymes responsible for miRNA 3′-terminal uridylation modifications. HCV uses microRNA-122 (miR-122), which is modified by 3’-end uridylation, as a proviral host factor to increase viral replication.^87^ The HCV core protein plays a role in reprogramming the cellular profile of miR-122 isomers by inhibiting germline development defective 2 (GLD-2), a noncanonical cytoplasmic poly(A) polymerase, leading to miR-122 destabilisation and increased viral production.^87^ Moreover, SARS-CoV-2 utilises the uridylate-specific endoribonuclease nonstructural protein 15 (Nsp15) to process its genome for efficient viral replication.^88^ On the other hand, another study has shown that terminal uridyltransferases 4 and 7 (TUT4/7)-mediated uridylation inhibits mouse hepatitis virus (MHV) and IAV transcript formation and viral replication.^89^ ^,^ ^90^

Uridylation has been shown to have antiviral effects within the context of innate immunity. The addition of uridine to the 3’ RNA terminus, catalysed by TUT4/7, can directly alter the genome by forming a poly(U) tag structure that enhances immune recognition of IAV vRNA via the host’s mRNA decay pathways.^90^ Uridylation modification in viruses plays a crucial role in their interactions with host cells by influencing RNA stability and degradation. However, the specific mechanisms by which uridylation affects viral replication and immune evasion remain largely unexplored. Additionally, the diversity of uridylation patterns across different viral families and their functional consequences are not well understood, highlighting the need for further research in this area. Understanding these aspects could lead to new therapeutic strategies against viral infections.

Antiviral therapy based on RNA modifications

Innovative strategies are increasingly focusing on epitranscriptomic modulators to enhance antiviral responses. This approach targets chemical modifications of RNA, which can significantly influence viral replication and the host’s immune response. By exploiting differences in epitranscriptomic profiles between viral and host RNA, researchers aim to develop RNA-targeting therapies that selectively inhibit viral infections.

Currently, the development of antiviral drugs primarily focuses on identifying viral proteins and structures as potential targets for various compounds and small molecules known as direct-acting antivirals (DAAs). DAAs, such as protease inhibitors, have been shown to influence specific RNA modifications critical for regulating antiviral responses. For example, 3-deazaadenosine, an m^6^A inhibitor, hinders IAV replication by depleting intracellular levels of the methyl donor S-adenosylmethionine (SAM).^91^ DAAs are particularly effective against DNA viruses, which exhibit a relatively low rate of spontaneous mutations. In contrast, their effectiveness against RNA viruses varies, necessitating independent evaluation for each DAA. Nonetheless, some DAAs have proven efficacy against RNA viruses, such as reverse transcriptase inhibitors for HIV^92^ and remdesivir, which targets the RNA polymerase of SARS-CoV-2.^93^ Overall, DAAs face challenges such as viral resistance, prolonged treatment durations, potential combined adverse reactions, and high costs.^94^

Another promising approach in antiviral therapy involves RNA interference (RNAi), including siRNAs and miRNAs. These molecules can be designed to specifically target vRNA and host factors involved in viral life cycles, leading to degradation of the viral genome and inhibition of replication. The programmability of siRNAs and miRNAs allows them to be tailored for various viral infections, making them a compelling therapeutic strategy. RNAi is activated upon the presence of dsRNA generated during viral replication, leading to the production of siRNAs that specifically target viral mRNAs for degradation, effectively blocking the replication process. Studies have shown that siRNAs targeting WNV significantly reduce viral protein expression and genomic RNA synthesis.^95^ Additionally, RNAi can target accessory genes that viruses possess, which contribute to their pathogenesis and ability to evade host defences. Silencing these genes through RNAi can disrupt viral latency and reduce replication; in HIV-1, for instance, siRNAs targeting various regions of the viral genome have demonstrated substantial reductions in virus production.^96^ However, many viruses have evolved mechanisms to counteract RNAi, including the expression of viral suppressors of RNA silencing (VSRs).^97^ An example is the DENV NS4B protein, which modulates the host’s RNAi pathway to favour its own replication by interfering with antiviral responses.^98^ RNAi serves as a crucial regulatory mechanism in both controlling viral replication and modulating immune responses in host cells. Additionally, eukaryotic RNAi pathways usually require small RNAs (rRNA, tRNA, lncRNAs, miRNA, and snRNA) to guide Argonautes, a family of proteins essential for regulating gene expression in eukaryotic cells.^99^ Crucially, small RNAs not only guide but also influence the activity of RNA modification enzymes on their substrate RNAs. Although evidence for this phenomenon is still limited, one notable example is small nucleolar RNA C/D Box 115 cluster (SNORD115), which directs 2’-O-methylation (Nm) modification to an ADAR2-mediated pre-mRNA editing site, thereby specifically interfering with ADAR2 activity.^100^

Natural compounds have emerged as promising antiviral regulators that target specific RNA modifications, playing a vital role in influencing various biological processes, particularly antiviral responses. For m^6^A, resveratrol may enhance antiviral responses by inhibiting m^6^A methylation and suppressing viral gene expression,^101^ ^,^ ^102^ ^,^ ^103^ while curcumin decreases host m^6^A levels, impacting RNA stability and translation.^102^ Additionally, quercetin acts as an inhibitor of METTL3, leading to decreased m^6^A levels and demonstrating antiviral effects against various RNA viruses.^104^ In the case of m^5^C, glycyrrhizin inhibits endocytic uptake of IAV and potentially suppresses its m^5^C methylation by reducing the activity of DNMTs in host cells,^105^ ^,^ ^106^ ^,^ ^107^ and curcumin is also believed to modulate m^5^C levels in host RNA by inhibiting DNMT1, contributing to its antiviral activity.^108^ ^,^ ^109^ Furthermore, for 2’-O-Me/Nm, thymol, found in thyme oil, may disrupt vRNA modifications,^108^ while ginsenosides derived from ginseng might influence RNA processes related to 2’-O-Me/Nm modifications.^110^ Overall, these natural compounds highlight the potential of targeting RNA modifications for therapeutic applications in viral infections and beyond.

In conclusion, the intersection of RNA modification research and antiviral drug development presents a promising frontier in virology. By targeting both vRNA modifications and host cellular mechanisms, new therapeutic strategies can be developed to effectively combat a wide range of viral infections. Continued exploration of these pathways will likely yield innovative treatments that leverage our understanding of RNA biology in the context of viral pathogenesis.

Conclusions and future directions

Epitranscriptomics, the study of post-transcriptional RNA modifications, has unveiled a new layer of gene expression regulation with profound implications for various biological processes, including virus pathogenesis and host immune responses. The dynamic landscape of RNA modifications, such as m^6^A, m^5^C, m^1^A, and 2’-O-me/Nm, plays a pivotal role in shaping the epitranscriptome and influencing gene expression at the post-transcriptional level. Studies have elucidated the complex interplay between RNA modifications and nucleolytic events, highlighting the intricacies of the epitranscriptome and its effects on RNA stability, translation, degradation, and host immune regulation.

Antiviral therapy based on RNA modifications faces several challenges that need to be addressed for future advancements. Viral adaptation poses a significant hurdle, as viruses can evolve rapidly, developing resistance to therapies targeting RNA modifications. Viral RNA modifications such as m^6^A, m^5^C, and pseudouridine play key roles in virus evolution by facilitating rapid adaptation to host environments.^9^ These modifications directly impact viral replication by promoting RNA stability and recruiting host factors that enhance replication efficiency, while also regulating translation through effects on ribosome binding and transcript processing.^18^ Additionally, codon usage bias varies among viruses and often adapts to match host tRNA abundances, optimising translation efficiency and viral fitness, which influences viral pathogenicity and cross-species transmission.^111^ Together, RNA modifications and codon usage shape viral evolution, replication, and protein synthesis in complex host-virus interactions.

Additionally, host toxicity is a concern, as some compounds may inadvertently affect host RNA processes, leading to undesirable side effects. For example, certain antiviral and anticancer agents, such as nucleoside analogues like 5-fluorouracil (5-FU), can be incorporated into host RNA, causing RNA damage that disrupts normal cellular functions and leads to cytotoxicity in human tissues.^112^ These off-target effects can manifest as side effects like peripheral neuropathy, cardiomyopathy, or cytopenia, which are linked to the disruption of key RNA processing pathways in host cells, including mitochondrial and nuclear RNA polymerases.^112^ Additionally, compounds that interfere with tRNA modification or aminoacylation can impair protein synthesis and cellular viability, further contributing to toxicity.^113^ Thus, while these drugs may be effective against viruses, their impact on host RNA biology must be carefully monitored to minimise harmful side effects.

Furthermore, there is a lack of comprehensive understanding of the complex roles that various RNA modifications play in both viral and host biology, which complicates the development of effective antiviral strategies. Addressing these challenges will be crucial for optimising antiviral therapies that leverage RNA modifications.

Looking ahead, the future of epitranscriptomic modifications holds exciting prospects for advancing our understanding of gene regulation and disease pathogenesis. High-throughput technologies and advanced sequencing methods are revolutionising the field, enabling researchers to decipher the epitranscriptomic landscape with unprecedented precision and depth. High-throughput sequencing technologies such as Illumina short-read, PacBio SMRT, and Oxford Nanopore long-read sequencing have revolutionised viral infection and virus-host interaction studies by enabling comprehensive viral genome sequencing and detection of intra-host variants.^114^ Viral RNA enrichment methods like dsRNA purification and tiling amplicon sequencing (PrimalSeq) improve sensitivity for low-abundance viruses, making it possible to identify variants that might otherwise remain undetected.^115^ Oxford Nanopore’s direct RNA sequencing allows detection of native vRNA modifications, providing insights into epitranscriptomic regulation of viral replication and immune evasion.^114^ Additionally, integrated profiling of viral and host transcriptomes reveals how host noncoding RNAs modulate viral replication and immune responses, advancing our understanding of virus-host dynamics.^114^ ^,^ ^115^ The identification of novel RNA modifications, the characterisation of their functional roles, and the exploration of their regulatory mechanisms are paving the way for groundbreaking discoveries in epitranscriptomics.

Understanding epitranscriptome regulatory mechanisms during viral infection significantly advances our knowledge of host responses by revealing how both host and vRNAs are dynamically modified to influence gene expression, immune evasion, and the outcome of infection. Viruses manipulate the host’s RNA modification machinery to reprogram the epitranscriptomic landscape, enhancing their own replication and helping them escape immune detection, while host cells also adjust their RNA modifications to mount effective antiviral responses. For example, modifications such as m^6^A on host or vRNAs can modulate the efficiency of viral genome translation, alter immune signalling, and impact the stability of both host and viral transcripts.^111^

This mechanistic insight opens new avenues for therapeutic development. By targeting specific RNA modification enzymes or pathways hijacked by viruses, it may be possible to inhibit viral replication or restore effective host immunity, providing a novel class of antiviral drugs.^111^ For instance, blocking the enzymes that install or remove key modifications could disrupt viral life cycles without directly targeting viral proteins, potentially reducing the risk of resistance.^116^

Leveraging advanced epitranscriptomic technologies, such as high-throughput sequencing and modification mapping, enables precise profiling of RNA modifications during infection.^111^ These tools can identify diagnostic biomarkers-unique modification patterns associated with specific viral infections or disease states-and guide the development of targeted therapies that modulate the epitranscriptome for therapeutic benefit. Ultimately, integrating these technologies with virology and immunology will foster innovative diagnostic and therapeutic solutions for viral diseases.
